# Covid-19 pandemic: from carnival masks to surgical masks

**DOI:** 10.1590/2175-8239-JBN-2020-0078

**Published:** 2020-08-28

**Authors:** Carmen Tzanno-Martins

**Affiliations:** 1 Grupo CHR, São Paulo, SP, Brasil.; 2 Sociedade Brasileira de Nefrologia, São Paulo, SP, Brasil.; 3 Sociedad Latinoamericana de Nefrología e Hipertensión, Departamento de Economía, Ciudad de Panamá, Panamá.; 4 International Society of Nephrology, Clinical Research Committee, Cranford, United States.

**Keywords:** Coronavirus Infections, COVID-19, Betacoronavirus, SARS-CoV-2, Pandemics, Dialysis, Nephrology, Hypertension, Comorbidity, Infecções por Coronavirus, Covid-19, Betacoronavirus, SARS-CoV-2, Pandemias, Diálise, Nefrologia, Hipertensão, Comorbidade

## Abstract

Given the high transmissibility of SARS-CoV-2, COVID-19 pandemic has a huge impact on our health system. Even in developed countries, strategic resources soon become insufficient. Although people over 60 and with comorbidities are at greater risk of developing severe forms, younger people may also require precious and scarce care. Hence, the World Health Organization recommend tests - PCR and serological tests - for detecting infected people on a large scale. The most common symptoms are fever, fatigue, dry cough, anorexia, myalgia, and dyspnea, with tomographic pulmonary findings being frequent even in asymptomatic cases. The Brazilian Society of Nephrology has published guidelines for the management of hypertensive, diabetic, dialysis, and transplant patients. In its alerts, care and precautions in dialysis units are also being detailed, both for the health team and for the patients. Although important renal manifestations are not yet evident in the admission of positive cases, recent studies with renal patients and performed in nephrology services are listed here. This pandemic lead us to learn from its progress in order to face new challenges in dialysis clinics, transplant services, and intensive care services.

## Resumo

Dada a elevada transmissibilidade do SARS-CoV-2, a pandemia de covid-19 causa enorme impacto no Sistema de Saúde. Mesmo em países do primeiro mundo, recursos estratégicos logo se tornam insuficientes. Ainda que pessoas acima de 60 anos e portadores de comorbidades, como hipertensão, corram maior risco de desenvolver formas graves, os mais jovens também podem requerer cuidados preciosos e escassos. Daí a Organização Mundial da Saúde recomendar testes - PCR e sorológicos - para detecção de infectados em larga escala. Sintomas mais comuns são febre, fadiga, tosse seca e dispneia, sendo frequente o achado tomográfico de manifestações pulmonares, mesmo em casos assintomáticos. A Sociedade Brasileira de Nefrologia tem divulgado diretrizes para manejo de pacientes hipertensos, dialíticos e transplantados. Em seus alertas, cuidados e precauções em unidades de diálise também estão sendo detalhados, tanto para a equipe de saúde como para os pacientes. Embora ainda não se evidenciem manifestações renais importantes na admissão dos casos positivos, são arrolados aqui estudos recentes com pacientes renais e realizados em serviços de nefrologia. Essa pandemia nos obriga a aprender com seu avanço, a fim de enfrentarmos novos desafios nas clínicas de diálise, nos serviços de transplante e de terapia intensiva.

The world has lived through many epidemics throughout history. Brazil has faced the epidemic of HIV, swine flu, dengue, and Zika. However, none of them impacted the health system like the covid-19 pandemic due to its high transmissibility and long recovery time.

On December 31, 2019, the World Health Organization (WHO) was alerted about cases of pneumonia caused by a new strain of Coronavirus in the city of Wuhan, Hubei province, People’s Republic of China. On January 7, 2020, China confirmed that it had identified the virus.^1^

Over the first two decades of this century, other coronaviruses have been identified, such as SARS-COV (Severe Acute Respiratory Syndrome) and MERS-COV (Middle East Respiratory Syndrome). However, we are facing a new pathogen responsible for the disease COVID-19, initially named 2019-nCOV and which, on February 11, 2020, received the definitive designation of SARS-CoV-2.^2^

On February 18, 48 hours after the first case of the disease was detected in Brazil, the team of researchers from the Institute of Tropical Medicine at the University of São Paulo, led by the Brazilian physician Ester Sabino, announced that they had completed the genome sequencing of the new coronavirus.^3^

The outbreak that began in China and, at first, was expected to be short-lived and restricted to Asia spread to the four continents, given its high transmissibility, especially in the centers with the highest demographic density. Thus, on March 11, 2020, WHO declared SARS-CoV-2 a pandemic.^1^

In the fight against the disease, strategic resources, such as individual protection equipment, ventilators, and intensive care beds, as well as the number of health professionals, have been insufficient even in developed countries.

As it is a new pandemic, we are learning from it at the same time as we face its spread. We learned that people over 60 years of age and those with comorbidities such as cardiovascular disease and hypertension are at greater risk of developing its most severe form.^4^

At the time of this writing, on March 27, 2020, we have confirmed 462.684 cases worldwide. However, this number may be underestimated, as a large proportion of asymptomatic people or with mild symptoms have not been tested. In addition, there may be false negatives. So far, there are 20.834 deaths worldwide, while in Brazil there are 3.417 confirmed cases. Of the 92 deaths, most (68) occurred in the state of São Paulo. 5

The WHO recommends testing for infection on a large scale, as it has been done in Germany, South Korea, Singapore, and Hong Kong. This strategy seems to work in these countries, as they have lower death rates secondary to coronavirus infection. Whoever presents symptoms and tests positive is isolated, blocking transmission. In this case, only symptomatic and positive individuals are isolated.^1^

The PCR test is the most sensitive method for diagnosis and is the gold standard. However, it requires more complex infrastructure. Its analysis period is long and it can be negative due to the drop in viral titer. Real time, faster tests are also being offered. Serological tests for the detection of IgM / IgG antibodies are ineffective for diagnosis in the acute phase, but have the advantage of being faster and simpler. Although useful for decision making, they are less sensitive than PCR and indicated for negative PCR cases that show symptoms of pneumonia for more than 10 days.^6^^,^ 7

The incubation period, in general, is up to 7 days. In descending order, the most common symptoms are fever, fatigue, dry cough, anorexia, myalgia, and dyspnea. Fever can be very low (<38 degrees) in 20% of cases. After the onset of symptoms, dyspnea may persist and SARS usually develops 8 days after onset, often requiring prolonged mechanical ventilation (20 days on average). The white blood cell count is usually normal, and lymphopenia is frequent in the most severe cases. CRP is elevated with normal procalcitonin. Liver enzymes are elevated in about 20% of cases. Changes in renal function at admission were observed in only 1.6% of patients.^4^^,^ 8

Pulmonary manifestations are common in the tomography exam, even in asymptomatic cases. The most frequent finding is ground-glass opacities. However, the image patterns are diverse, change during evolution, and can be similar to infection by H1N1, bacterial, or respiratory syncytial virus, and therefore a definitive diagnosis cannot be reached by imaging tests alone.^9^

## What Nephrologists Need To Know About Covid-19

The Brazilian Society of Nephrology (BSN) has used communication through digital media and has endeavored to keep periodic updates since the beginning of the pandemic. Through these updates, guidelines are released for clinical management of hypertensive outpatients, patients with rare diseases, in acute and chronic dialysis, adults, pediatric and transplanted ones. All recommendations are updated as more knowledge about the new disease is acquired and according to international and national scientific guidelines. Recommendations are available with free access on the SBN website (www.sbn.org.br).

One of the first controversies to arise was regarding the prescription and maintenance of ACE (angiotensin converting enzyme) inhibitors and ARB (angiotensin receptor blocker). A large number of hypertensive, cardiac, diabetic, and proteinuric patients use these drugs, which are common in our daily practice. The ACE-2 receptor is present in type 2 pneumocytes in the lungs, but it is also common in the myocardium, arteries, kidneys, and intestines.^10^ The SARS-Cov-2 virus binds to ACE2 receptors and is internalized, initiating viral replication,^11^ causing its downregulation with elevation of angiotensin II and increased pulmonary vascular permeability and, consequently, the appearance of severe acute respiratory syndrome (SARS).^12^ As these drugs cause an increase in the number of ACE2 receptors, a hypothesis was raised that patients using these drugs could be more susceptible because of the increased targets of the virus. However, no study supports this hypothesis, and the American Heart Association and the Brazilian Society of Nephrology do not recommend the suspension of these drugs.

Smokers have an increased expression of ACE-2 receptors and therefore also have an increased risk of susceptibility to the virus^13^. However, in a meta-analysis of preliminary results from Covid-19 patients in China, there was no correlation between smoking and disease severity. 14

It is not yet known exactly how the virus affects the kidneys. However, it seems to be a multifactorial process. One of the hypotheses is a direct action on the renal cells. Recent studies have shown a predominance of tubular injury, possibly because the tubules are the site with the highest expression of ACE-2 in the kidney. The SARS-CoV-2 nucleocapsid-protein was found in the cells of the renal tubules. However, it is not yet clear whether these cells would also serve as a reservoir for the virus.^15^ Another cause of kidney damage results from inflammation secondary to lymphocyte activation (lymphocytes express ACE-2) with high production of cytokines.

In addition, the activation of the complement and coagulation cascade, greater platelet aggregation, and high oxidative stress may affect the kidney. Microthrombi, hemosiderin deposits, and endothelial lesions were found in necropsies of patients who died with COVID-19. However, these findings often showed no clinical correlation, that is, patients had unchanged renal function tests.

Many critically ill patients develop evidence of microangiopathy in other organs. Some may have abdominal pain and hematuria suggesting renal infarction.^16^ The presence of proteinuria could be explained by viral replication in podocytes. Also, some cases of collapsing glomerulopathy have been described.^17^ A recent study evaluating the prognosis and renal manifestations in 333 patients with COVID-19 reported that 75.4% of them had proteinuria, hematuria, and acute kidney injury. The average duration of symptoms and signs was 12 days and about half of the patients with renal failure recovered their function in 3 weeks. Patients with renal impairment had a worse prognosis, with higher mortality.^18^ However, the long-term renal impact and how these patients should be monitored is not yet known.

## Potential Impact On Patients And Professionals

A study carried out in Wuhan, China, with about 1,000 patients, reveals that renal manifestations do not seem to be important on admission. Only 0.7% of patients had chronic kidney disease, creatinine was elevated in 1.6% of patients, acute kidney injury in 0.5%, and only 0.8% of patients required renal replacement therapy. The frequency of acute kidney injury and use of RRT was 4.3% and 5.2% in patients with more severe conditions.^4^ Another multicenter Chinese study with 193 patients reported that 59% had proteinuria, 44% hematuria, and only 10% had elevated creatinine^19^.

Data from a single dialysis unit at Renmin Hospital in Wuhan University, between January and February 2020, revealed new coronavirus infection of 37 out of 230 hemodialysis patients (16%) and 4 staff members out of a total of 33 (12%). Most had mild or moderate symptoms. Seven patients died in the observation period, 6 with COVID-19^20^.

Another paper on precautionary measures carried out in Wuhan in dialysis clinics cites unpublished data from He F and Xu G showing that only 10% of patients on a dialysis program in the city of Wuhan and 6.4% of the medical staff had COVID-19^21^. This percentage was even lower in the region of Lombardy, Italy, according to data presented by Dr Brunori, President of the Italian Society of Nephrology. Among 3,318 patients on a chronic hemodialysis program and representing 75% of patients on a chronic dialysis program in the area, only 8% (206/3318) were positive for COVID-19 and 1.8% (62/3318) died^22^.

Another important issue concerns the care and precautions that must be taken in dialysis units. The recommendations of the Brazilian Society of Nephrology are in line with those of other international medical societies. Measurement of temperature on arrival in the unit by both staff and patients, investigation of symptoms, especially the most common ones, isolation of symptomatic patients or with a history of contact in separate shifts or rooms, examination of symptomatic patients whenever possible, education and training on the use of personal protective equipment, especially masks, gloves and glasses or face shields, as well as frequent hand hygiene with soap and water are some of the recommended measures.

The most severe conditions and the highest mortality are observed among the elderly and adults with comorbidities such as hypertension, lung diseases, and other chronic diseases. Patients with more severe conditions may have renal failure and multiple organ failure. Positive cases should be isolated until the symptoms improve (usually two weeks) and released after two negative tests collected at 24-hour intervals.

The pandemic can be analyzed from a technical-assistance, humanitarian and spiritual point of view, or from an economic perspective. This editorial did not intend to address all these facets and was restricted to medical aspects. However, we will have a new world order after this pandemic, which will certainly affect our practice. Home-office, telemedicine, investments in infrastructure and research in health, and economic recession are now being experienced in an intense and globalized way.

I hope that learning from this pandemic will endure and prepare us for the next ones, as they will certainly come.
